# Omalizumab: An Optimal Choice for Patients with Severe Allergic Asthma

**DOI:** 10.3390/jpm12020165

**Published:** 2022-01-26

**Authors:** Serafeim Chrysovalantis Kotoulas, Ioanna Tsiouprou, Eva Fouka, Athanasia Pataka, Despoina Papakosta, Konstantinos Porpodis

**Affiliations:** Pulmonary Department, Medical School, Aristotle University of Thessaloniki, “G. Papanikolaou’’ General Hospital, Exohi, 57010 Thessaloniki, Greece; akiskotoulas@hotmail.com (S.C.K.); evafouka@auth.gr (E.F.); patakath@yahoo.gr (A.P.); depapako@gmail.com (D.P.); kporpodis@yahoo.gr (K.P.)

**Keywords:** omalizumab, severe allergic asthma, IgE

## Abstract

Omalizumab is the first monoclonal antibody that was globally approved as a personalized treatment option for patients with moderate-to-severe allergic asthma. This review summarizes the knowledge of almost two decades of use of omalizumab to answer some important everyday clinical practice questions, concerning its efficacy and safety and its association with other asthma-related and drug-related parameters. Evidence suggests that omalizumab improves asthma control and reduces the incidence and frequency of exacerbations in patients with severe allergic asthma. Omalizumab is also effective in those patients in reducing corticosteroid use and healthcare utilization, while it also seems to improve lung function. Several biomarkers have been recognized in predicting its efficacy in its target group of patients, while the optimal duration for evaluating its efficacy is between 16 and 32 weeks.

## 1. Introduction

Asthma is a heterogeneous disease with different identifiable clusters of demographics, clinical and/or pathophysiological characteristics, often referred to as asthma phenotypes [1]. The most common asthma phenotype is allergic asthma, which counts for up to 80% of childhood asthma and more than 50% of adult asthma cases [2,3]. Nowadays, an improved understanding of the pathophysiology of asthma and the identification of different phenotypes led to the development of personalized, phenotype-guided treatments [4,5,6].

Omalizumab, a recombinant humanized monoclonal antibody which specifically binds to the C epsilon3 domain of immunoglobulin (Ig) E was marketed in the dawn of the 21st century and was the first among many of such treatments [7]. Omalizumab is currently suggested in patients aged six years or older with difficult-to-treat to severe persistent allergic asthma according to the Global Initiative for Asthma (GINA) guidelines, who fulfill one or more of the following criteria: sensitization to inhaled allergen(s) on a skin prick testing or specific IgE.; and body weight within a local dosing range, and more than a specified number of exacerbations within the last year, despite a daily high dose of inhaled corticosteroids, plus a long-acting inhaled beta2-agonist [1,8,9].

This review consolidates the experience of two decades of using this personalized treatment for patients with severe allergic asthma, answering some questions from daily clinical practice about the effectiveness of omalizumab in improving asthma symptoms, reducing corticosteroid use, reducing exacerbation rate and healthcare utilization. The purpose of this review is to describe omalizumab’s safety and its association with biomarkers and lung function and to define the duration of its administration to evaluate its effectiveness.

## 2. Omalizumab’s Efficacy on Asthma Symptom Improvement

Several tools are available for monitoring asthma control, such as validated questionnaires (asthma control test (ACT) [10], an asthma control questionnaire (ACQ) [11], and an asthma quality of life questionnaire (AQLQ) [12], etc. [10,11,12,13,14,15,16].

Ayres et al. proved that out of a total of 312 patients with poorly controlled, severe, allergic asthma, those who were treated with omalizumab plus the best standard care experienced a significant improvement in asthma symptom control compared to the control group, where the best standard care was offered (Table S1) [17]. In another randomized controlled trial, which included 400 asthmatic patients, Bousquet et al. demonstrated that asthma control, measured by ACQ score was significantly improved both at 16 and at 32 weeks in patients who received omalizumab compared to controls (Table S1) [18]. Moreover, the results of several early clinical trials [19,20,21,22,23,24], as well as of later ones, [25,26] indicated symptom improvement in asthmatic patients, both children and adults, who received omalizumab, compared to the controls, although those results were not always statistically significant. Nevertheless, a systematic review with meta-analysis which combined these studies concluded that in a total of 3429 participants, asthmatic patients who received omalizumab exhibited a statistically significant improvement not only in asthma control scales but also in quality-of-life scales, such as AQLQ.; compared to the controls [27]. The same findings were also demonstrated by another systematic review with meta-analysis, which included three randomized, double-blind, placebo-controlled studies, that enrolled 1412 patients with moderate or severe allergic asthma [28].

Apart from clinical trials, there are also several observational, every-day clinical practice studies which also evaluated the efficacy of omalizumab in asthma symptom control. Barnes et al. demonstrated that both asthma control, measured by ACT and quality of life, measured by AQLQ.; were significantly improved in asthmatic patients after the initiation of omalizumab both at 16 and at 52 weeks [29]. Similar results were also published by Deschildre et al., who used GINA criteria to assess asthma control in children with asthma after the initiation of omalizumab (Table S1) [30]. Researchers found that the initiation of omalizumab in patients with severe allergic asthma led to a significant improvement in both daily and nightly symptoms as well as in different asthma symptom scales such as AQLQ.; mini-AQLQ and ACT [31,32,33]. On the other hand, Nopp et al., in a very small-scale study, reported that 8 out of 18 asthmatic patients who were treated with omalizumab for 6 months still exhibited asthma symptom improvement 3 years after the discontinuation of omalizumab [34]. However, another study which included 943 patients with asthma reported a significant reduction in (a) daily symptoms, (b) activity limitation, (c) night symptoms, (d) night awakenings due to asthma and (e) the need for reliever medication, after the initiation of omalizumab both at 12 and at 24 months. In the same study, the findings from both ACT and ACQ scales were also significantly improved [34,35].

## 3. Effectiveness of Omalizumab in Reducing Corticosteroid Use

Alongside standard treatment with ICS.; patients often need to be treated with oral corticosteroids (OCS) to achieve better asthma control [35,36,37,38,39,40,41,42,43,44]. Novel therapy strategies aim to reduce the use of corticosteroids to eliminate possible adverse effects [45,46,47,48,49,50,51,52,53].

Busse et al. found that ICS administration was reduced by 75% in patients with severe asthma 28 weeks after the initiation of omalizumab, which was significantly greater than the 50% reduction in the control group (Table S1) [24]. Similar results about ICS reduction were also reported by another clinical trial at 32 weeks after omalizumab initiation (57.2% of the patients who received omalizumab vs. 43.3% of the placebo group, *p* < 0.05) [21]. Moreover, the effect of omalizumab on ICS reduction was predicted by the Archimedes asthma model for US patients aged 12 and older with moderate-to-severe persistent allergic asthma [54,55,56,57,58]. Apart from ICS dosage reduction, omalizumab seems to be even more effective in OCS dosage reduction and/or discontinuation [56,59,60,61,62,63]. The beforementioned effects of omalizumab in corticosteroid use have also been confirmed by four systematic reviews with meta-analyses [8,27,64,65].

In addition to clinical trials, the impact of omalizumab on corticosteroids use in severe allergic asthma has also been demonstrated by several observational real-life studies. The initiation of omalizumab resulted in significant reduction in ICS dosage in several studies [30,32,33,66,67], while in another study, its discontinuation led to a significant increase in prescribed ICS dosage [34]. Furthermore, the initiation of omalizumab had the same effect in OCS usage, as it led to significant OCS dosage reduction or discontinuation in several studies [29,35,68,69,70,71,72], while in another study both ICS and OCS reduction was observed in patients with severe allergic asthma who were treated with omalizumab for seven years [73].

## 4. Efficacy of Omalizumab in Reducing the Rate of Asthma Exacerbations

Severe exacerbations may occur even in patients with mild or well-controlled asthma symptoms as a patient’s risk of exacerbations may be independent of the level of symptom control [73,74,75]. More importantly, exacerbations were proved fatal on many occasions [76,77,78,79,80]. Therefore, a successful therapeutic strategy should prevent asthma exacerbations.

Omalizumab resulted in the reduction of the asthma exacerbation rate in numerous clinical trials during the last two decades, both in adults and in children [17,18,19,20,21,22,23,24,25,26,55,60,81,82,83,84]. In two clinical trials, the exacerbation rate was significantly reduced in the omalizumab group (between 35–45% reduction) compared to the control group [18,21]. Ayres et al. reported 1.12 exacerbations per patient per year in the omalizumab group, which was significantly lower than the 2.86 exacerbations per patient per year in the control group (Table S1) [17]. Solèr et al., in one of the first clinical trials of omalizumab which included 546 participants, reported that both the number of exacerbations per patient during the stable-steroid phase (0.28 in the group who received omalizumab vs. 0.66 in the placebo group) as well as during the steroid-reduction phase (0.36 in the group who received omalizumab vs. 0.75 in the placebo group) and the number of patients needed to treat in order to avoid an exacerbation were significantly lower in the omalizumab group compared to the control group (35 in the group who received omalizumab vs. 83 in the placebo group during the steroid-reduction phase/43 in the group who received omalizumab vs. 81 in the placebo group during the steroid-reduction phase) (Table S1) [22]. On the other hand, there are also clinical trials in which the asthma exacerbation rate was not significantly reduced in the omalizumab group compared to the control group [57,85,86]. However, several systematic reviews with meta-analyses have concluded that omalizumab significantly reduces the asthma exacerbation rate compared to a placebo [27,28,64,87]. Moreover, another randomized controlled trial has proved that omalizumab is more effective in preventing asthma exacerbations in fall compared to an inhaled corticosteroid boost [88].

Apart from clinical trials, omalizumab has also been shown to be effective in the reduction of the asthma exacerbation rate in numerous observational real-life studies [29,30,31,32,33,34,35,66,68,73,89,90,91,92,93,94,95,96,97,98]. Barnes et al. and Deschildre et al. reported a significant reduction in asthma exacerbations after the initiation of omalizumab from 3.67 to 1.70 per patient per year and from 4.40 to 1.25 per patient per year, respectively (Table S1) [29,30]. Two more studies reported a significant reduction in asthma exacerbations per patient per year after the initiation of omalizumab (from 5.00 to 0.63 and from 5.70 to 1.90, respectively) [32,68]. Three more studies reported a significant reduction in the asthma exacerbation rate between 62% and 82% [31,33,66]. Nopp et al. found that 16 out of 18 patients who discontinued omalizumab still had fewer exacerbations during nights, even 3 years after the drug withdrawal [34]. The effect of omalizumab’s discontinuation on the increase of exacerbations was confirmed by two recently published studies [99,100].

## 5. Omalizumab’s Efficacy on Healthcare Utilization

Omalizumab has been shown to be effective in reducing the rate of emergency care visits and hospitalizations [77,79,101,102,103,104,105,106,107,108,109,110,111,112,113,114]. A clinical trial reported that patients treated with omalizumab suffered from 4.92 of such events per year compared to the 9.76 of such events per year in the control group [17]. Humbert et al. showed that, at 28 weeks, omalizumab significantly reduced emergency care visits and hospitalizations compared to the control group (0.24 vs. 0.43 and 0.125 vs. 0.25, respectively) [19]. Moreover, Bousquet et al. have proved a significant reduction in emergency care visits by 60% and in hospitalizations by 67% in the omalizumab group compared to the control group [18].

Real-life studies have demonstrated a significant reduction in emergency care visits and hospitalizations after the initiation of omalizumab which varied between 51–90.8% and 28–95%, respectively [30,31,56,66,115,116,117]. Other studies also reported a significant reduction in hospitalizations from 1.07–5.93 per patient per year to 0.10–2.75 per patient per year and in emergency care visits from 1.13–1.52 per patient per year to 0.29–0.46 per patient per year after the initiation of omalizumab [29,61,96]. Furthermore, Braunstahl et al., in the context of “eXpeRience registry”, which included 943 patients with asthma, reported a reduction in the unscheduled asthma-related visits in a healthcare provider from 6.2 per patient per year to 1.0 per patient per year and to 0.5 per patient per year at 12 and 24 months, respectively (Table S1) [35]. Other studies have demonstrated that after four-to-five years from the initiation of omalizumab almost no patient needed an asthma-related hospitalization [32,94,117], while Molimard et al. reported that the reduction in emergency care visits and hospitalizations after the initiation of omalizumab is irrespective of the reduction or the cessation of corticosteroids (Table S1) [68]. In addition to hospital admission, omalizumab also appears to significantly reduce the duration of hospitalization and the cost per hospital stay [118,119,120].

## 6. Safety Outcomes

Several asthma medications, and especially corticosteroids, present a wide spectrum of potentially serious adverse effects, particularly OCS in prolonged administration [45,46,47,48,49,121,122], but also in shorter use [50,123]. Omalizumab has been studied thoroughly during the last two decades and it seems to be a well-tolerated add-on treatment. Adverse events include anaphylaxis, malignancy, and symptoms such as serum sickness and eosinophilic conditions [8].

Clinical studies provide data on the safety and tolerability profile of omalizumab therapy in adults with asthma, urticaria, food allergies and allergic rhinitis [17,18,21,24,26,54,82,85,86,124,125,126,127,128,129,130,131,132,133]. The most common adverse events that were attributed to omalizumab in various clinical trials were local reactions to the injection site (i.e., redness and swelling), urticaria, erythema, headache, ear symptoms, sinusitis, nasopharyngitis, bronchitis, asthma, infections such as upper respiratory tract infection, influenza, pneumonia, gastrointestinal infections (appendicitis and intestinal geohelminth infection) and serum disease with or without anaphylaxis (Table S2) [19,25,113,133,134,135,136,137,138,139,140]. No adverse effects were revealed by hematology and biochemistry laboratory measurements [138,141]. There was no adverse effect regarding electrocardiographic data [138,141]. Omalizumab seems to be related with a lower risk for anaphylaxis compared to other monoclonal antibodies for asthma treatment, but when anaphylaxis occurred, patients with asthma who were treated with omalizumab seemed to be at greater danger for life-threatening reactions than those who are treated with omalizumab for urticaria [142]. Most adverse events are mild to moderate and usually do not lead to drug discontinuation [20,83,139,143,144]. A systematic review with meta-analysis of 3429 patients revealed that the patients who received omalizumab had a comparable percentage of side effects and serious side effects with the control group (1.3% vs. 1.5% and 3.8% vs. 5.3%, respectively). Patients in the omalizumab group exhibited a significantly higher percentage of local reactions in the injection site compared to the placebo group (19.9% vs. 13.2%, *p* = 0.002) [27]. There were no other significant differences between the two groups in separate adverse events such as urticaria, anaphylaxis, cardiovascular events, malignancies, or deaths [27]. A disproportionality analysis of the WHO’s VigiBase pharmacovigilance database showed that omalizumab may be associated with a significantly higher risk of malignancies. However, this result refers to a disproportionality analysis and it should be interpreted with caution, as retrospective and prospective cohorts are needed to confirm the findings [145]. Moreover, cases of death are rare and not directly attributable to omalizumab [138].

Observational studies were conducted to assess the adverse effects of this monoclonal antibody. Overall, the safety profile was consistent with the current label. Newly recognized adverse effects such as malignant neoplasms, relapsing herpes labialis, pyrexia, fatigue, chest pain, nausea, arthralgia, myalgia, type 1 hypersensitivity, angioedema, metrorrhagia and abortion were reported (Table S2) [146,147,148,149,150,151,152,153].However, the relative risk ratio for these adverse events seems to be very low [32,70,73,92,154,155,156,157,158,159,160,161,162,163,164]. In several observational studies the percentage of adverse events varies between 2.5% and 32.9% [31,33,35,66,71,94,95,97,115,165,166], the percentage of serious adverse events varies between 0.0% and 16.1% [30,33,66,71,95,97,115,167] and the rate of drug discontinuation due to an adverse event varies between 0.0% and 12.5% [30,33,35,94,115,165,166,167]. Long-term studies may need to adequately assess the risk of malignancy.

## 7. Association of Biomarkers with Outcomes of Omalizumab-Treated Patients

Several biomarkers have been identified and studied with respect to responsiveness of drug therapy in T2 high asthma, such as: total IgE.; periostin, blood eosinophils and FeNO.

Serum levels of total IgE were found increased 16 and 28 weeks after the initiation of omalizumab in patients with asthma [24], while they were decreased after seven years of use in another study [73]. Lowe et al. analyzed the data from previously published studies and found that once long-term observations were included, the models allowing IgE production to decrease fitted with their analysis [168]. Free IgE levels were decreased by 89–99% after the initiation of omalizumab [22,24], and this reduction was associated with a lower rate of exacerbations after two years of treatment [169]. Omalizumab has also been found to decrease the sensitivity to CD antigens of the responsible allergens after long-term use irrespective of total or specific IgE levels [34]. Apart from IgE.; which is an obvious target for omalizumab, other biomarkers such as T-helper-type-2 (Th2) inflammation biomarkers such as blood and/or sputum eosinophils and fraction of exhaled nitric oxide (FeNO) have been linked with omalizumab [92,93,170]. Serum periostin was also studied as a biomarker, as it is generated in response to IL-13 and serves as a marker of Th2-related inflammation [170,171]. There are some studies which have not found an association between these biomarkers and the response to omalizumab [92,93,170]. However, the majority of the studies demonstrate such an association, with the high levels of blood eosinophils and FeNO and, to a lesser degree, of serum periostin to be associated with higher response rates to the treatment with omalizumab in terms of symptom and exacerbation control, OCS usage, hospital admissions and lung function [90,169,171,172,173,174,175]. Moreover, histological and immunological studies of the airways have shown that omalizumab reduces the mucosal concentration of IgE and the tissue eosinophil cell density [176]. Furthermore, the use of FeNO measurement to specify omalizumab responders may decrease the expected per-patient cost [177]. Those results have led the American Thoracic Society (ATS) and the European Respiratory Society (ERS) to recommend blood eosinophil count and FeNO as biomarkers with a high value for the increased response to omalizumab and periostin as a biomarker with a low value [178]. Nevertheless, novel histological, serological and molecular studies have identified additional biomarkers which are predictive of a response to omalizumab such as bronchial protein galectin-3 [179,180], serum pregnancy-associated plasma protein-A (PAPP-A) [181], serum C4Ma3, a degradation marker of the inflammation-protective collagen 4 (COL4A3) [182] and CD3E antigen of immune cells [178].

## 8. Omalizumab and Lung Function

Patients with severe allergic asthma often experience a significant decline in lung function. Despite the fact that some studies have not found a significant improvement in forced expiratory volume in 1 s (FEV1) after the initiation of omalizumab [118,166,183,184,185,186,187,188,189], the majority of the studies have exhibited a significant improvement in FEV1 after the initiation of omalizumab in asthmatic patients both as a whole [17,29,32,33,59,60,67,73,81,86,119,152,153,158,162,173,190,191,192,193,194,195,196,197,198], and in specific groups such as children [199], obese patients [200], patients with fixed airway obstruction [201], patients with various co-morbidities [202], and even non-atopic patients with asthma [87]. Similar improvements after the initiation of omalizumab have also been observed in forced vital capacity (FVC) [33,203], FEV1/FVC ratio [59,73], peak expiratory flow (PEF) [19,86,204,205,206,207], small airway flows (FEF25-75) [199,208], and airway resistances [209,210,211,212]. Interestingly, omalizumab also seems to improve hyperinflation due to air trapping measured by the residual volume to total lung capacity (RV/TLC) ratio [213]. Furthermore, it has been demonstrated that omalizumab reduces the response to the methacholine and adenosine 5’-monophosphate (AMP) challenge test by increasing the provocative concentration required to produce a 20% fall in FEV1 and increases the tolerability of asthmatic patients to longer allergen exposure [211,214,215,216]. Finally, FEV1 has served as a predictor of the response to omalizumab with lower values leading to better response rates [60,217,218,219,220,221].

## 9. Optimal Duration of Treatment

It is crucial to determine a certain duration of omalizumab administration to assess its efficacy. Various criteria have been proposed, such as the characteristics of patients (age etc.) and biomarkers (IgE levels, FeNO etc.).

The minimum period for omalizumab effectiveness assessment was four weeks in one study [24]. According to most studies, this period varies between 16–20 weeks to 26–32 weeks [18,19,20,21,22,24,29,30,31,32,33,63,66,68,93]. There are also studies which evaluated omalizumab for an even longer period, although these studies were either early clinical trials [17], or observational studies which lasted longer mainly because they tried to evaluate omalizumab’s safety profile [29,30,32,33,34,35,73,93]. Ayres et al., Barnes et al., Deschildre et al. and Humbert et al. evaluated omalizumab for or up to 52 weeks [17,29,30,93]. Braunstahl et al. and Tzortzaki et al. assessed omalizumab for a period between one and two years and one and four years, respectively [33,35]. Moreover, other researchers used even longer periods, such as three, four, seven or up to nine years [32,34,73]. Two systematic reviews with meta-analyses reported that the studies which they included, evaluated omalizumab for time periods which varied between 16 and 58 weeks [27,28]. Nevertheless, the evidence suggests that the optimal duration of omalizumab administration in order to assess its efficacy varies between 16 and 32 weeks.

## 10. Conclusions

Omalizumab has been consistently proved effective in improving asthma symptom control irrespective of the scale that was used to measure its efficacy. It is also effective in reducing corticosteroid usage both inhaled and systematic, thus minimizing the catastrophic adverse effects of corticosteroids in patients with asthma. Omalizumab also reduces the exacerbation rate in patients with severe allergic asthma and the healthcare utilization. Overall, omalizumab presents a rather safe profile, even though long-term cohort studies are necessary to evaluate its relative risk, if there is any, for some serious adverse effects such as malignancies and abortions. Omalizumab also seems to improve various parameters of lung function. Finally, several biomarkers can be used to assess its efficacy or the need for its discontinuation, with the optimal duration of administration for this evaluation being set at 16–32 weeks.

## Supplementary Materials

The following supporting information can be downloaded at: https://www.mdpi.com/article/10.3390/jpm12020165/s1, Table S1: Efficacy of omalizumab in patients with severe allergic asthma; Table S2: Common adverse events of the use of omalizumab in asthmatic patients.
