# Supplementary material for: Omalizumab: An Optimal Choice for Patients with Severe Allergic Asthma
Source: J Pers Med. 2022 Jan 26;12(2):165. doi: 10.3390/jpm12020165 (PMC8878072; doi:10.3390/jpm12020165)
Supplement: Supplementary file 1 [file jpm-12-00165-s001.zip › jpm-1442573-supplementary.pdf]

Table S1. Efficacy of omalizumab in patients with severe allergic asthma.

Study	Design	Number of patients	Main outcomes
**Busse et al. (2001)**	phase III, double-blinded, placebo-controlled trial	525	fewer asthma exacerbations per subject with omalizumab vs placebo (0.28 vs 0.54, p=0.006 during stable steroid phase and 0.39 vs 0.66, p=0,003 during steroid reduction phase)
**Solèr et al. (2001)**	multi-center, randomized, double-blind, placebo-controlled, parallel-group study	546	omalizumab group vs placebo group: 58% and 525 fewer exacerbations per patients during stable-steroid and steroid-reduction phase (p<0,001), comparable overall incidence of adverse events between groups (p=0,504)
**Ayres et al. (2004)**	randomized, open-label, multicenter, parallel-group study	312	annualized mean number of asthma-deterioration related incidents/mean exacerbation rates with BSC alone vs with omalizumab: 9.76 vs 4.92 (p<0.001)/ 2.86 vs 1.12 (p<0.001)
**Holgate et al. (2004)**	meta-analysis of three randomized, double-blind, placebo-controlled studies	1412	mean significant exacerbation rate per patient-year: 1.56 with placebo vs 0.69 with omalizumab, p=0,007, omalizumab vs placebo: improvement from baseline in PEFR (p=0,026), overall AQoL (p=0,042), mean nocturnal (p=0,007) and total (p=0,011) asthma symptoms
**Humbert et al. (2005)**	double-blind, parallel-group, multicenter study	419	omalizumab reduced severe asthma exacerbation rate vs placebo (0.24 vs 0.48,p=0,002) and emergency visit rate (0.24 vs 0.43, p=0,038), omalizumab significantly improved sthma-related quality of life, morning peak expiratory flow and asthma symptom scores
**Brusselle et al. (2009)**	open-label, multicenter, pharmaco-epidemiologic study	158	>82% improvement in total AQLQ scores of > or = 0.5 points (p<0,001), >91% were exacerbation-free (p<0,001) at 16 weeks vs >84% improvement in total AQLQ scores of > or = 0.5 points (p<0,001), >65% were exacerbation-free (p<0,001) at 52 weeks
**Bousquet et al. (2011)**	randomized, open-label, multicenter, parallel-group study	400	omalizumab-treated patients: improvement in exacerbation rates (p<0.001), severe exacerbation rates (p<0.05), hospitalizations (p<0.05), total emergency visits (p<0,05), ACQ score (p<0.001)
**Barnes et al. (2013)**	10-center retrospective observational study	147	34% reduction in total OCS prescription at 12 months, improvement in mean FEV_1% at 16 weeks (62,94 vs 70,98, p<0,01), reduction in healthcare utilization at 12 months (p<0,05)
**Deschildre et al. (2013)**	1-year real life multicenter survey	104	decrease in exacerbations (4.4 to 1.25 per patient after 1 year), decrease in hospitalizations by 88.5%, mean improvement of lung function: 4.9% pred. for FEV_1 95% Cl, p<0,05) and 9.5% pred. for FEF_{25-75} (95% Cl, p<0,05)
**Braunstahl et al. (2013)**	international, single-arm, open-label, observational study	943	no clinically significant or severe clinically significant asthma exacerbations higher at 12 months (82.4% and 95.8%) and 24 months (81.9% and 95.6%) vs pre-treatment period (4.9% and 27.4%), improvement in activity limitation (41.7%

			at 24 months vs 46.6% at 12 months, p<0,05) and in rescue medication use (49.8% at 24 months vs 54.1% at 12 months, p<0,05)
**Molimard et al. (2014)**	observational, descriptive, cross-sectional, retrospective study	61	loss of asthma control in 34 patients (55.7%) with a median interval between discontinuation and loss of control of 13.0 months (mean 20.4 ± 2.6 [95% CI: 8.3-28.1])
**Sposato et al. (2016)**	multi-center, observational study	105	improvement in ACT values (p<0.001;comparing pre-post) in each group (pre values: 15[IQR:12-18]; 14 [IQR:10-16]; 15 [IQR:12-16]; post-values: 24[IQR:22-25]; 21 [IQR:20-23]; 20 [IQR:18-22]; measured in young, middle-aged and elderly subjects), reduction in exacerbations and ICS treatment after omalizumab in each group (p<0,001)
**Iribarren et al. (2017)**	observational study	7836	omalizumab-treated patients had a higher rate of CV/CBV serious adverse events (13.4 per 1,000 person years [PYs]) than did non-omalizumab-treated patients (8.1 per 1,000 PYs), ATE rates per 1,000 PYs were 6.66 (101 patients/15,160 PYs) in the omalizumab cohort and 4.64 (46 patients/9,904 PYs) in the non-omalizumab cohort
**Ke et al. (2018)**	retrospective observational cohort study	1564	asthma-related medication use decreased from the preindex to the postindex periods (oral corticosteroids, p < 0.001; ICSs, p< 0.001; LABAs, p = 0.009; ICS-LABA combination, p<0.001; leukotriene modifiers, p< 0.001), the proportion of patients with any asthma exacerbations decreased by 33.6% (p< 0.001)
**Al-Ahmad et al. (2018)**	4-year observational study	65	ICS/LABA dose significantly reduced from 65 (100%) to 25 (38.5%) after 4 years of treatment (p < 0.001); ACT scores significantly increased from 15 ± 3 at baseline to 23 ± 3 (p < 0.001) and FEV1 level from 55.6 ± 10.6 to 76.63 ± 10.34 at year 4
**Oliveira et al. (2018)**	non-interventional, prospective study	32	at 12 months of omalizumab: improvement in BMI, number of exacerbations, rescue medication, disease control and lung function (p<0,05)

Table S2. Common adverse events of the use of Omalizumab in asthmatic patients.

**Adverse Effects**	
Local reactions to the injection site (i.e., redness, swelling)	45%
Viral infections	23%
Upper respiratory tract infections	20%
Sinusitis	16%
Headache	15%
Anaphylactic reactions	0.09 to 0.2%
